# Supplementary material for: Selective control of fcc and hcp crystal structures in Au–Ru solid-solution alloy nanoparticles
Source: Nat Commun. 2018 Feb 6;9:510. doi: 10.1038/s41467-018-02933-6 (PMC5802822; doi:10.1038/s41467-018-02933-6)
Supplement: Supplementary file 1 — Supplymentaly Information [file 41467_2018_2933_MOESM1_ESM.pdf]

Supplementary Information

Selective control of fcc and hcp crystal structures in Au–Ru solid-solution alloy nanoparticles

Quan Zhang[1], Kohei Kusada[1, *], Dongshuang Wu[1], Tomokazu Yamamoto[2,3], Takaaki Toriyama[3], Syo Matsumura[2,3,4], Shogo Kawaguchi[5], Yoshiki Kubota[6] & Hiroshi Kitagawa[1,4, *]

[1]*Division of Chemistry, Graduate School of Science, Kyoto University, Kitashirakawa-Oiwakecho, Sakyo-ku, Kyoto 606-8502, Japan*

[2]*Department of Applied Quantum Physics and Nuclear Engineering, Kyushu University, 744 Motooka, Nishi-ku, Fukuoka 819-0395, Japan*

[3]*The Ultramicroscopy Research Center, Kyushu University, Motooka 744, Nishi-ku, Fukuoka 819-0395, Japan*

[4]*INAMORI Frontier Research Center, Kyushu University, Motooka 744, Nishi-ku, Fukuoka 819-0395, Japan*

[5]*Japan Synchrotron Radiation Research Insitute (JASRI), SPring-8, 1-1-1 Kouto, Sayo-cho, Sayo-gun, Hyogo 679-5198, Japan*

[6]*Department of Physical Science, Graduate School of Science, Osaka Prefecture University, Sakai, Osaka 599-8531, Japan*

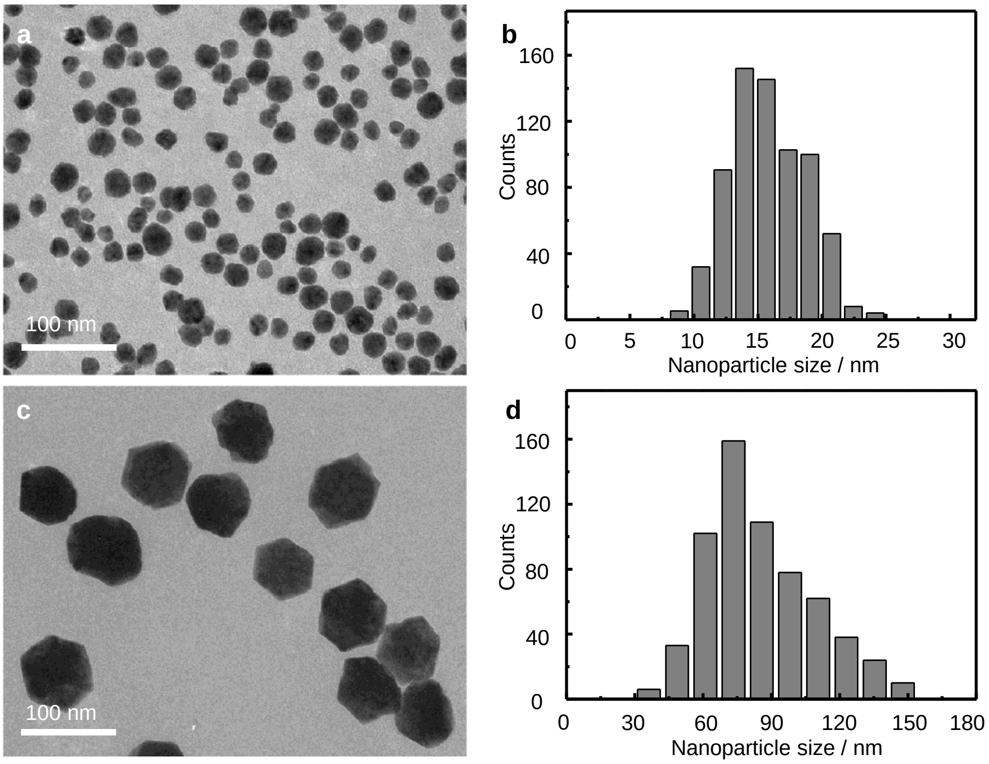

Supplementary Figure 1. (a) TEM image of the synthesized fcc-$AuRu_3$ NPs. (b) Size distributions of the nanoparticles in (a). (c) TEM image of the synthesized hcp-$AuRu_3$ NPs. (d) Size distributions of the nanoparticles in (c).

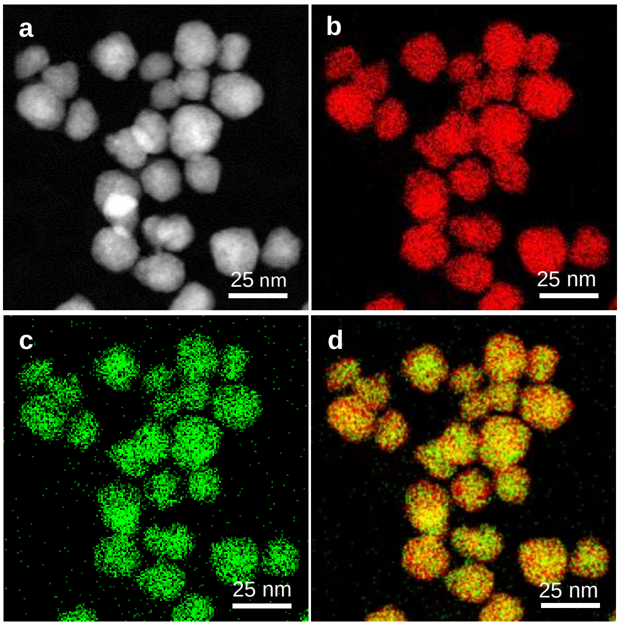

Supplementary Figure 2. (a) Low magnification HAADF-STEM image of fcc-$AuRu_3$ NPs. (b) Ru-L STEM−EDX map of (a). (c) Au-M STEM−EDX map of (a). (d) Overlay image of (b) and (c) (red, Ru; green, Au).

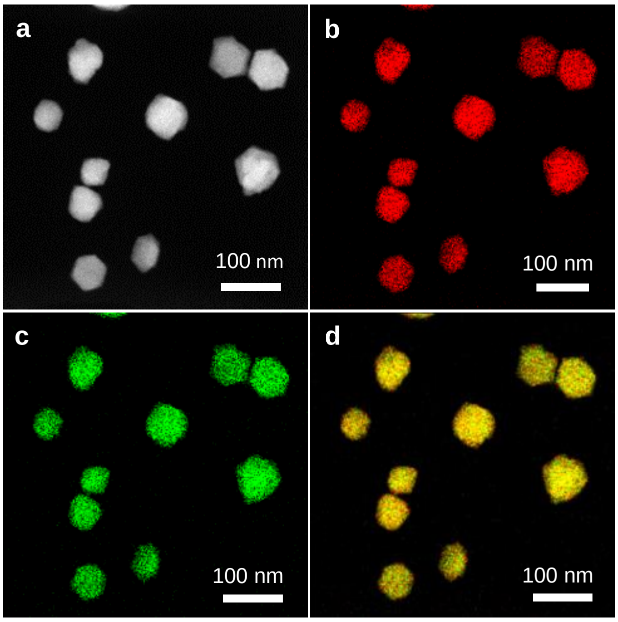

Supplementary Figure 3. (a) Low magnification HAADF-STEM image of hcp-$AuRu_3$ NPs. (b) Ru-L STEM−EDS map of (a). (c) Au-M STEM−EDX map of (a). (d) Overlay image of (b) and (c) (red, Ru; green, Au).

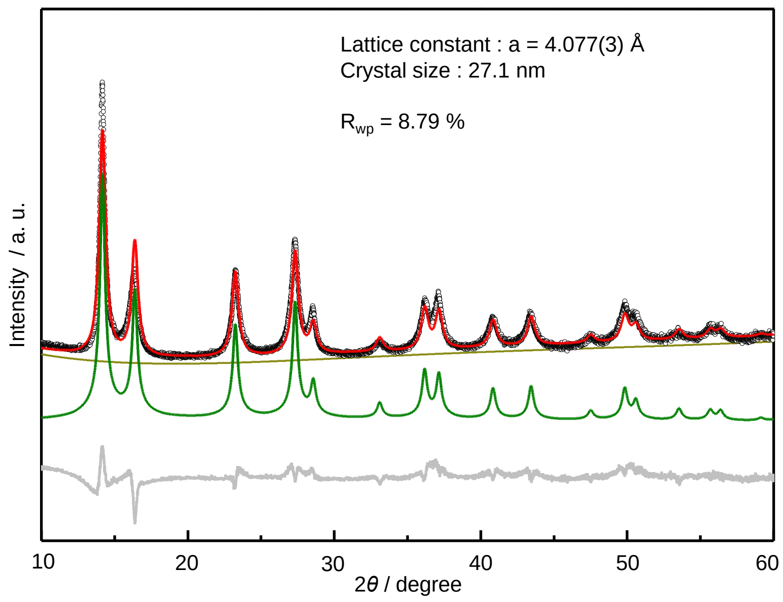

Supplementary Figure 4. Rietveld refinement for Au NPs. The diffraction patterns are shown as black circles. The calculated patterns are shown as red lines. The difference profile, the background profile, and the fitting curves of the fcc components are shown as grey, brown and green lines, respectively.

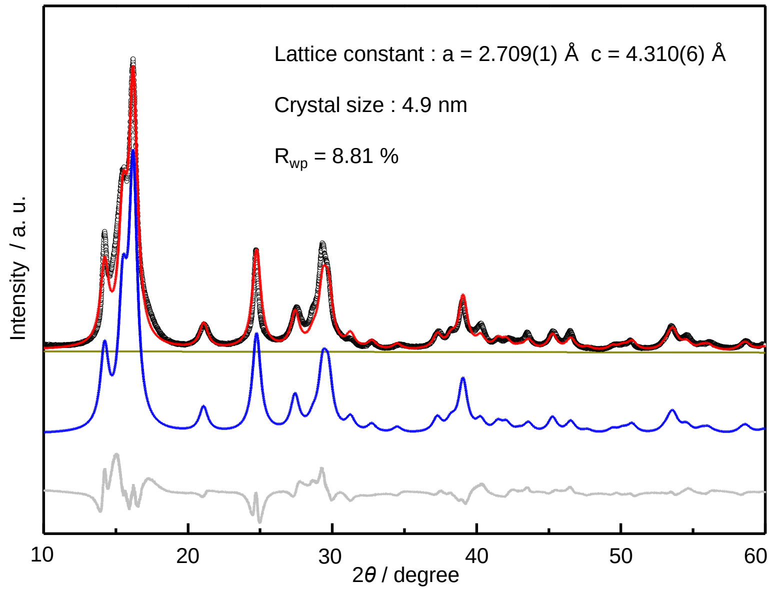

Supplementary Figure 5. Rietveld refinement for Ru NPs. The diffraction patterns are shown as black circles. The calculated patterns are shown as red lines. The difference profile, the background profile, and the fitting curves of the hcp components are shown as grey, brown and blue lines, respectively.

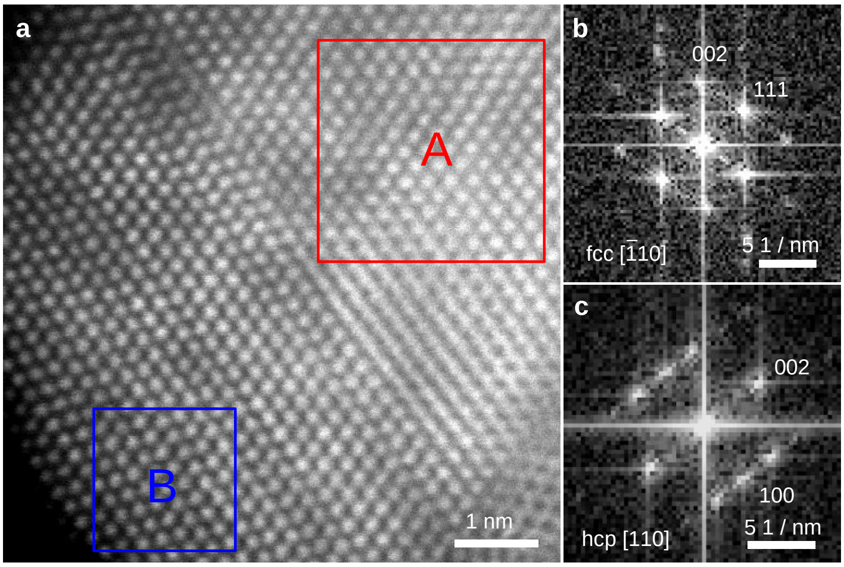

Supplementary Figure 6. (a) Low magnification HAADF-STEM image of the fcc-$AuRu_3$ NP. (b) The corresponding FFT pattern of the red square region A in (a). (c) The corresponding FFT pattern of the blue square region B in (a).

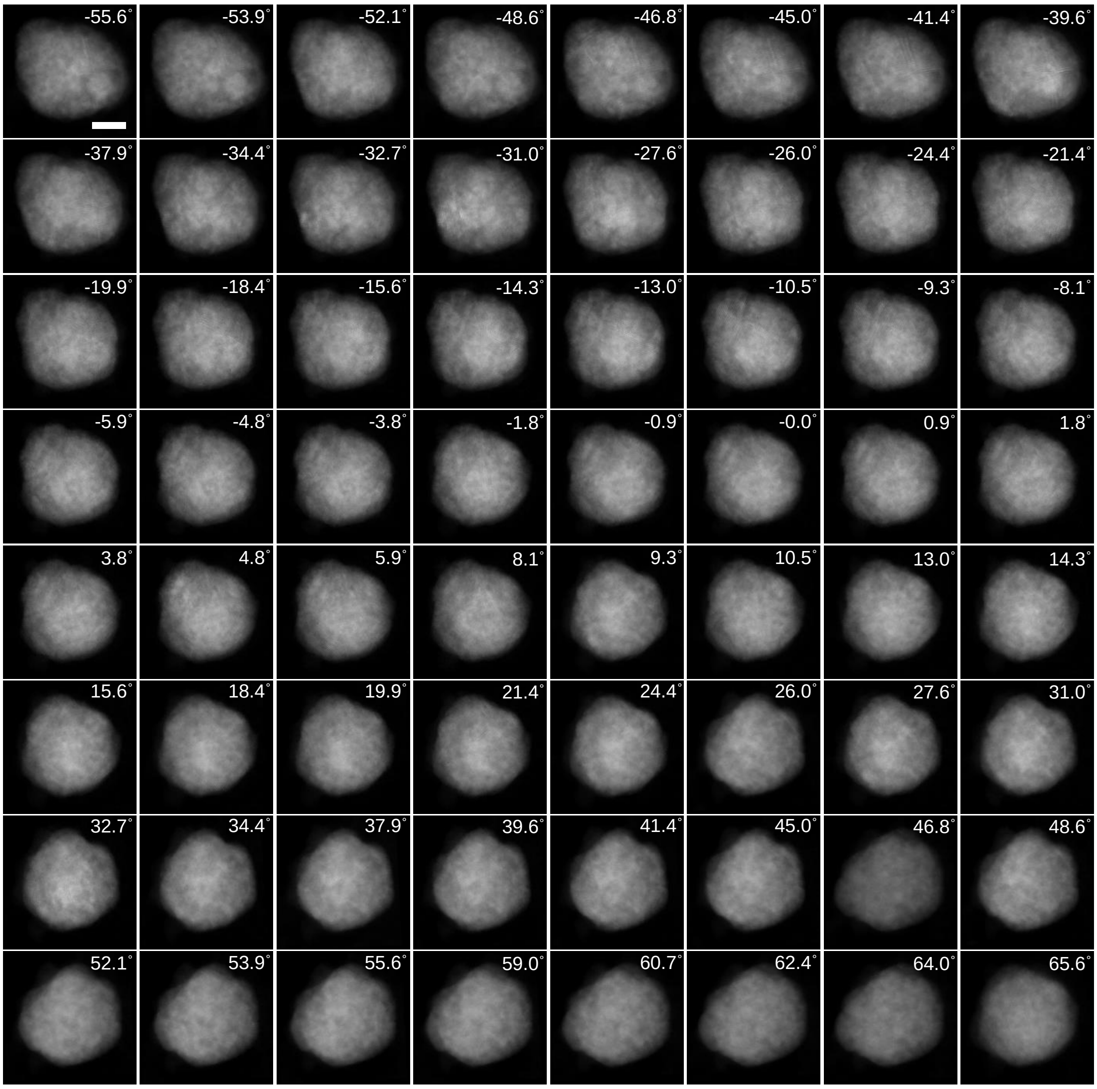

Supplementary Figure 7. Experimental tilt series obtained from a fcc-$AuRu_3$ NP. 64 projection images with a tilt range from -55.6˚ to +65.6˚ were measured using a JEM-ARM200F operated at 120 kV. The scale bar is 5 nm.

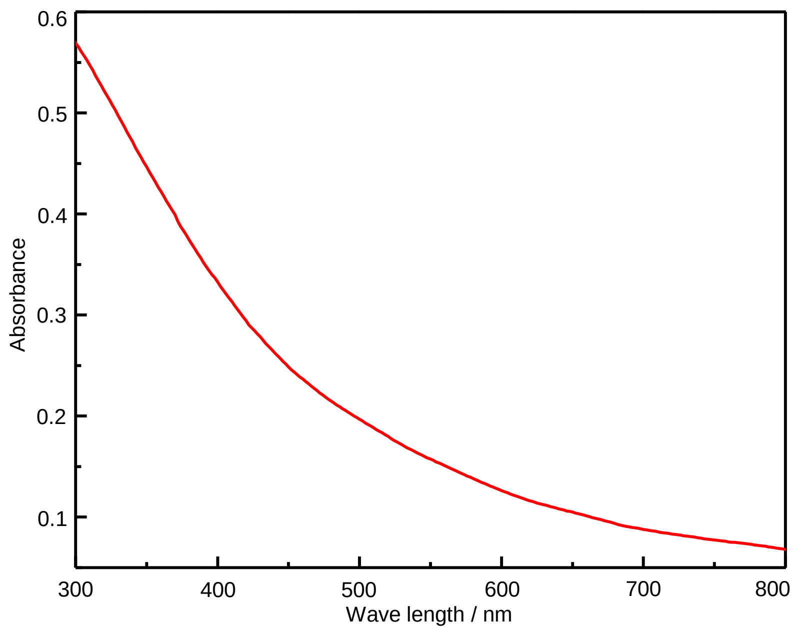

Supplementary Figure 8. UV-Vis spectrum of Ru NPs in the EG solution. The concentration of Ru NPs is 0.04 mg ml^{-1}.

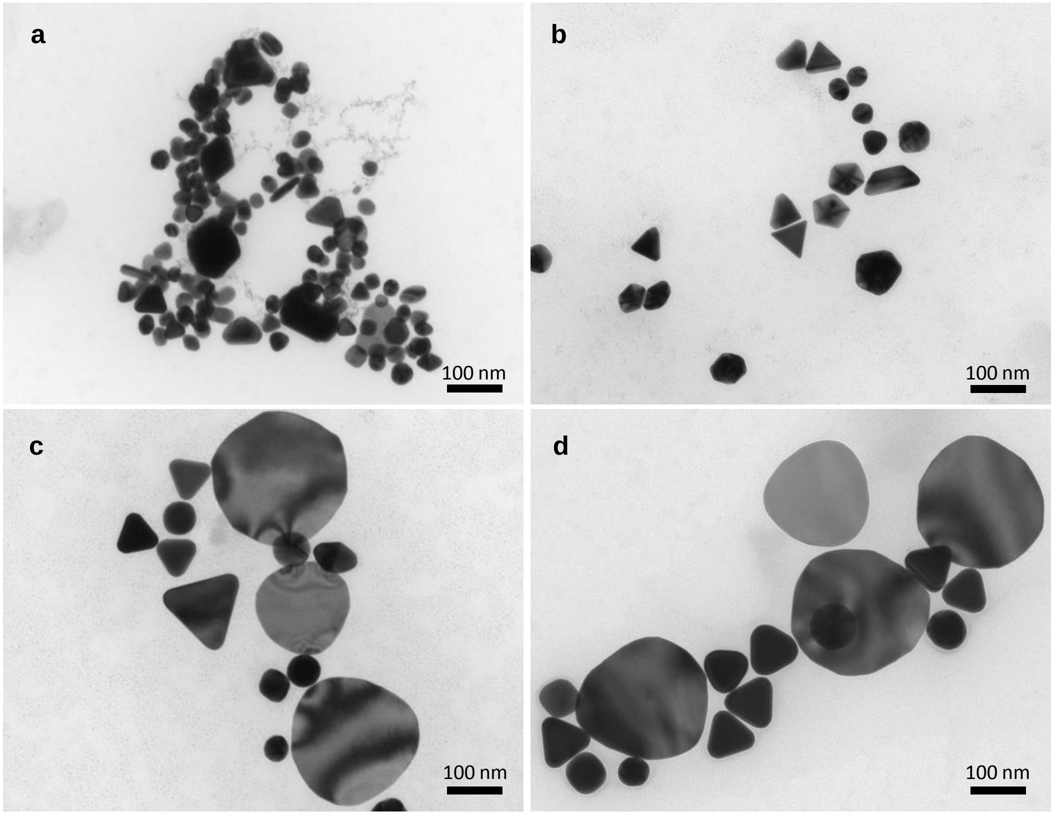

Supplementary Figure 9. TEM images of Au NPs at different reaction time during the reduction process of $HAuBr_4$ with CTAB in DEG. (a) 90 s, (b) 120 s, (c) 180 s, (d) 240 s.

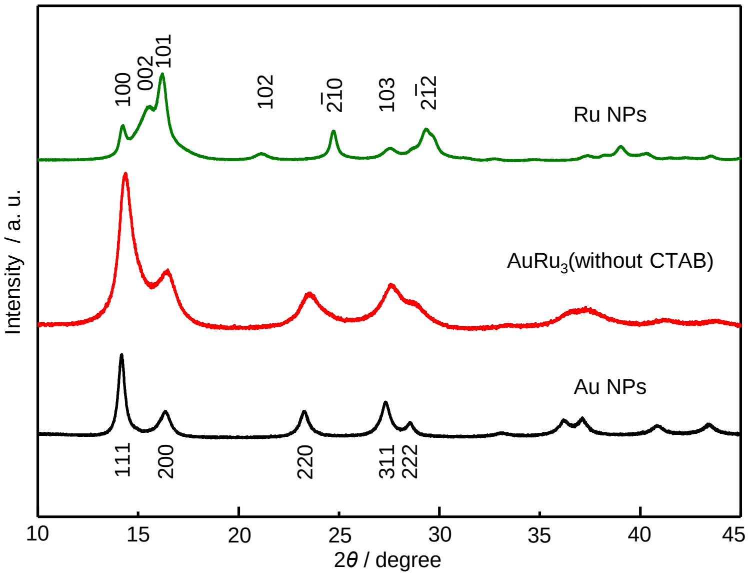

Supplementary Figure 10. Synchrotron XRD patterns of $AuRu_3$ NPs prepared using $HAuBr_4$ and $RuCl_3$ as metal precursors without using CTAB at 303 K. The radiation wavelength was 0.57865(1) Å.

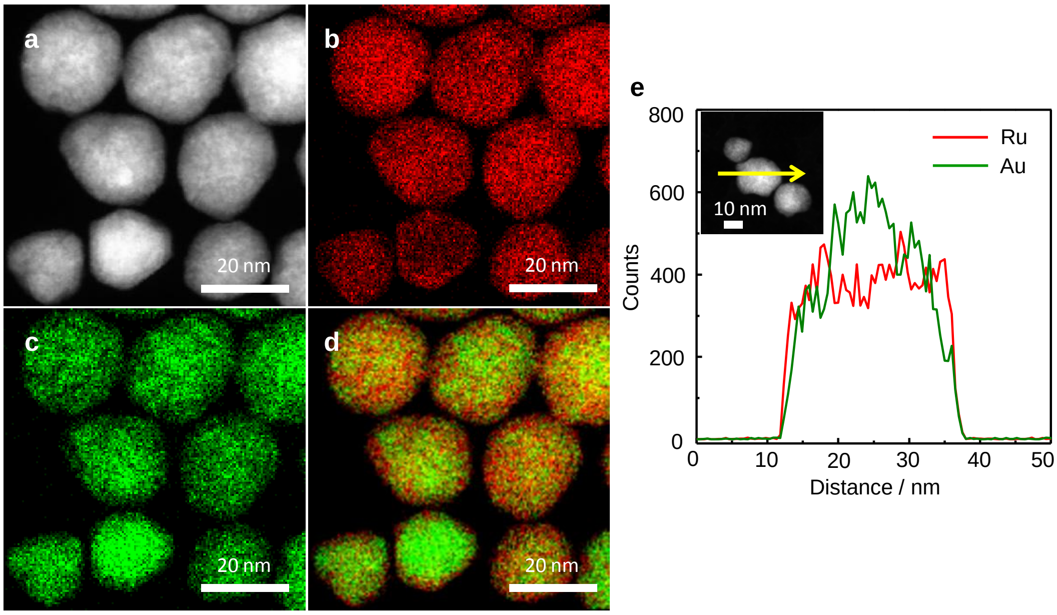

Supplementary Figure 11. (a) HAADF-STEM image of $AuRu_3$ NPs (synthesized using $HAuBr_4$ and $RuCl_3$ as metal precursors without using CTAB), (b) Ru-L STEM-EDX map and (c) Au-M STEM-EDX map. (d) Overlay image of (b) and (c) (red, Ru; green, Au). (e) EDX line profiles of $AuRu_3$ NPs across the NP along the arrow shown in the inset figure.

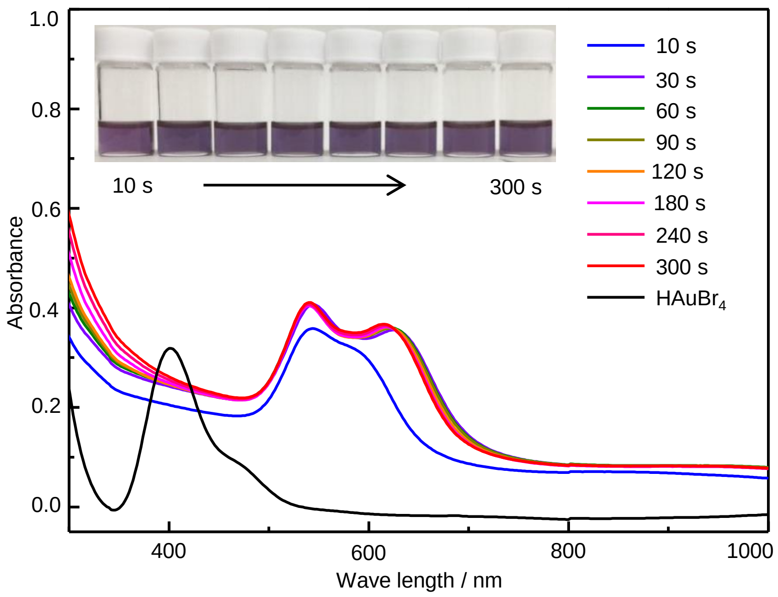

Supplementary Figure 12. UV-Vis spectra of $HAuBr_4$ in DEG with increasing reaction time. Insert photo shows the colour change during this process.

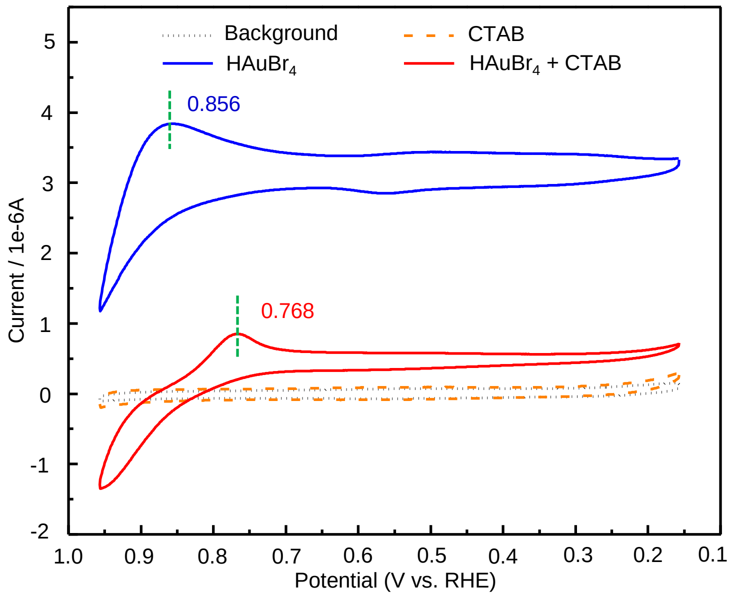

Supplementary Figure 13. Cyclic voltammograms of $HAuBr_4$ and $HAuBr_4$ with CTAB. The cyclic voltammograms were measured in a 0.05 mmol L^{-1} sulfuric acid aqueous solution at 0.02 V s^{-1}.
