# Supplementary material for: Selective control of fcc and hcp crystal structures in Au–Ru solid-solution alloy nanoparticles
Source: Nat Commun. 2018 Feb 6;9:510. doi: 10.1038/s41467-018-02933-6 (PMC5802822; doi:10.1038/s41467-018-02933-6)
Supplement: Supplementary file 3 — Description of Additional Supplementary Files [file 41467_2018_2933_MOESM3_ESM.pdf]

Description of Additional Supplementary Files

File Name: Supplementary Movie 1

Description: 3D reconstruction showing the overall view of the fcc-AuRu NP.

File Name: Supplementary Movie 2

Description: 3D reconstruction showing the layer view of the fcc-AuRu NP.
